# Free Fatty Acids and Endotoxins Synergically Induce Pyroptosis in Bovine Hepatocytes

**DOI:** 10.3390/metabo16010053

**Published:** 2026-01-08

**Authors:** Dan Li, Yuan Tian, Lei Tian, Hang Yu, Le Zhang, Song Wang, Changsheng Lei, Pin Long, Tao Peng, Lei Liu, Yingfang Zhou

**Affiliations:** 1 College of Veterinary Medicine, Hunan Agricultural University, Changsha 410128, China; 2 College of Animal Science and Technology, Hunan Biological and Electromechanical Polytechnic, Changsha 410128, China

**Keywords:** NEFA, LPS, pyroptosis, NLRP3, Caspase-1, bovine hepatocytes

## Abstract

**Background/Objectives**: Elevated circulating non-esterified fatty acids (NEFAs) are closely associated with hepatic inflammatory injury in dairy cattle, simultaneously with the entry of lipopolysaccharide (LPS) into the liver. This study aimed to investigate the synergistic effects of NEFAs and LPS on pyroptosis in bovine hepatocytes. **Methods**: Primary bovine hepatocytes were allocated into control, NEFA, NEFA + LPS, NEFA + LPS + Caspase-1 inhibitor, and NEFA + LPS + NLRP3 inhibitor groups. Levels and activation of pyroptosis-related markers (NLRP3, ASC, Caspase-1, GSDMD, IL-18 and IL-1β) were measured. **Results**: NEFAs alone upregulated these markers in a dose-dependent manner. Compared to NEFAs alone, NEFA + LPS co-treatment significantly enhanced levels of the markers, increased IL-1β secretion, and promoted NLRP3/Caspase-1 co-localization and Caspase-1activity. Notably, these effects of NEFA + LPS were attenuated by the NLRP3 or Caspase-1 inhibitors. Similar results were obtained when repeating the experiments in carcinoma HepG2 cells. Also, a random liver section from the subclinical ketotic cows displayed a higher fluorescence intensity of NLRP3 and Caspase-1 and stronger co-localization than that from a healthy cow. **Conclusions**: NEFAs and LPS synergistically contribute to pyroptosis in bovine hepatocytes by enhancing NLRP3 inflammasome assembly and subsequent Caspase-1 activation, providing a potential target for mitigating hepatic injury.

## 1. Introduction

During the transition period, dairy cows frequently experience a pronounced state of negative energy balance (NEB), which induces fat mobilization and subsequently elevates circulating concentrations of non-esterified fatty acids (NEFAs). These NEFAs are primarily taken up by the liver, where they undergo complete/partial oxidation, esterification into triglycerides or export as part of very low-density lipoproteins [1]. However, this metabolic overload of NEFAs can impair immune function and heightens the susceptibility to metabolic disorders (e.g., ketosis and fatty liver) and infectious diseases (e.g., mastitis and endometritis) [2]. Ultimately, these conditions compromise milk production, reduce overall efficiency, and, due to increased culling rates and treatment costs, inflict significant economic losses on the dairy industry.

Lipopolysaccharide (LPS), a major component of the outer membrane of Gram-negative bacteria, can be released into the bloodstream [3]. Because of the prevalence of Gram-negative bacteria within the bovine rumen and intestine, cattle naturally possess a significant reservoir of endotoxins [4]. Feeding high-concentrate diets to high-yielding dairy cows during the transition period (aiming to correct the NEB) frequently results in a reduction in ruminal pH [5]. This decline in pH can lead to the mortality of Gram-negative bacteria and compromise the integrity of the ruminal epithelial barrier [6]. Consequently, substantial quantities of LPS are released, which then traverse the compromised barrier and enter the bloodstream. Additionally, dairy cows are vulnerable to postpartum contamination of the uterine cavity by *Escherichia coli* and other pathogens. This condition facilitates the translocation of LPS from the uterine lumen into the systemic circulation [7].

Once in the portal vein, LPS travels to the liver [8], a vital organ in the absorption and detoxification of LPS [9,10]. The inflammatory role of LPS has been well-documented in various bovine cell types, including hepatocytes [11,12,13]. However, despite the co-occurrence of circulating NEFAs and LPS in dairy cows during the transition period, few studies have investigated the cooperative role of LPS and NEFAs in bovine pathophysiology, especially in hepatocytes.

Pyroptosis is a pro-inflammatory form of programmed cell death. It is commonly initiated through the canonical pathway, which involves NLRP3 inflammasome-dependent Caspase-1 activation [14,15]. The canonical NLRP3 inflammasome is a multi-protein complex comprising NLRP3, the adaptor protein ASC, and pro-Caspase-1. Upon sensing danger signals, this complex assembles and promotes the auto-cleavage and activation of Caspase-1 [16]. Active Caspase-1 then cleaves the precursors of IL-18 and IL-1β and simultaneously cleaves gasdermin D (GSDMD). The N-terminal fragment of GSDMD forms pores in the cell membrane, leading to cell lysis and pyroptosis [17]. Both NEFAs and LPS cause inflammatory injury in bovine cells [18,19]. Whether NEFAs further lead to pyroptosis in bovine hepatocytes and whether LPS aggravates this effect remains unclear.

We hypothesized that NEFAs and LPS synergistically activate the assembly of the NLRP3 inflammasome and lead to pyroptosis in bovine hepatocytes. We tested this hypothesis in primary calf hepatocytes and human hepatocellular carcinoma cells. The results of the current study may provide an additional perspective to prevent or alleviate hepatic inflammatory injury in dairy cows during the transition period.

## 2. Materials and Methods

### 2.1. Preparation of NEFAs

All fatty acids were from Sigma-Aldrich (St. Louis, MO, USA) and the NEFAs were prepared as previously described [20]. The NEFA stock solution (52.7 mM) consisted of palmitoleic acid (C16:1, P9417, 2.8 mM), oleic acid (C18:1, O1008, 22.9 mM), linoleic acid (C18:2n-6, L1376, 2.6 mM), palmitic acid (C16:0, P0500, 16.8 mM), and stearic acid (C18:0, S4751, 7.6 mM). The NEFA stock solution was diluted in 0.1 M KOH at 60 °C and its pH was adjusted to 7.4 with 1 M HCl. Before use, the NEFA solution was complexed with 2% BSA (A1130, Gentihold, Beijing, China) and then filter-sterilized (0.22 μm).

### 2.2. Hepatocyte Culture and Treatment

Primary hepatocytes were isolated from three healthy, 1-day-old Holstein female calves, each weighing between 30 and 40 kg, using the collagenase IV perfusion technique as previously described [20]. Each isolation was processed and treated independently, serving as an independent biological replicate (*n* = 3) for all subsequent experiments. Cells from different calves were not pooled. Initially, the caudate lobe of the liver was carefully isolated and rinsed thoroughly to remove any blood residues. The liver was subsequently perfused through its vasculature with perfusion solution A (pH 7.2–7.4, 37 °C), comprising 140 mM NaCl, 10 mM HEPES, 6.7 mM KCl, 0.5 mM EDTA, and 2.5 mM glucose, at a flow rate of 50 mL/min for 15 min. This was followed by perfusion with solution B (140 mM NaCl, 30 mM HEPES, 6.7 mM KCl, 5 mM CaCl_2_, and 2.5 mM glucose) under the same conditions for 3 min, until the perfusate was clear. Subsequently, a collagenase IV solution (0.2 g/L in perfusion solution B) was introduced at a flow rate of 20 mL/min until the perfusate became turbid, indicating sufficient tissue digestion. The digestion process was terminated by adding 100 mL of fetal bovine serum (FBS; FB15015, Clark Bioscience, Richmond, VA, USA). Following the removal of the liver capsule, blood vessels, adipose tissue, and connective tissue, the digested liver tissue was mechanically disaggregated and filtered sequentially through 150 µm and 75 µm meshes. The isolated hepatocytes were washed twice with a basic medium at 4 °C prior to resuspension in an adherent medium, specifically DMEM/F12 basic medium (C11995500BT, Gibco Life Technologies, Carlsbad, CA, USA) supplemented with 10% fetal bovine serum, 1 µM insulin, 1 µM dexamethasone, and 10 µg/mL vitamin C. The hepatocytes were then seeded into 6-well plates at a density of 1 × 10^6^ cells/mL (2 mL per well), and incubated at 37 °C in a 5% CO_2_ atmosphere. After 4 h of incubation, the medium was replaced with a complete medium containing 10% FBS, and refreshed daily thereafter.

HepG2 cells, obtained from iCell Bioscience Inc. (Shanghai, China), were cultured in MEM medium (C12571500BT, Gibco Life Technologies, Carlsbad, CA, USA) supplemented with 10% fetal bovine serum and 1% penicillin–streptomycin (15140-122, Gibco Life Technologies, Carlsbad, CA, USA) at 37 °C in a humidified 5% CO_2_ atmosphere. Upon reaching approximately 70% confluence, the cells were used for subsequent experimental treatments, structured into three distinct sections.

**Figure 1:**
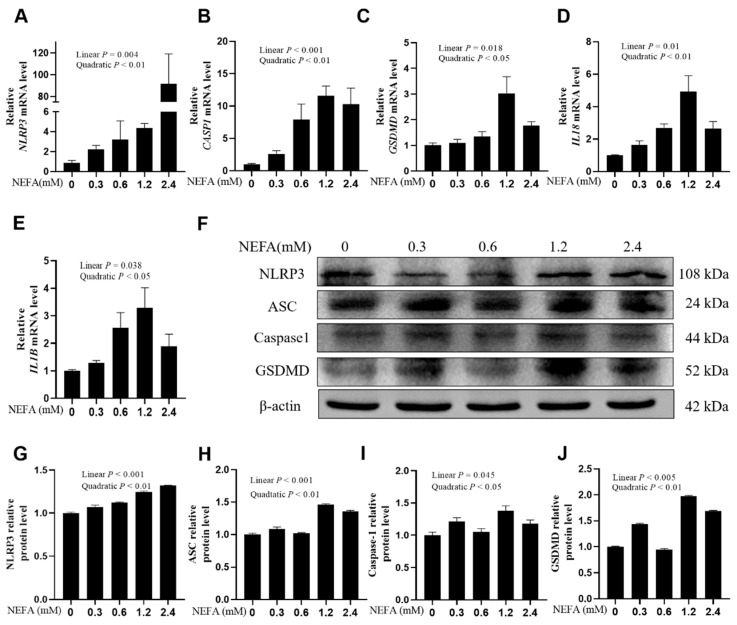
Effects of high NEFAs on expression levels of NLRP3, Caspase-1, GSDMD, IL-18 and IL-1B in dairy cow hepatocytes. (**A**–**E**) The mRNA relative expression of *NLRP3*, *CASP1*, *GSDMD*, *IL18* and *IL1B* was measured via real-time quantitative PCR with *ACTB* and *GAPDH* as the house keeping genes. (**F**–**J**) The relative protein levels of NLRP3, ASC, Caspase-1 and GSDMD was measured via western blotting. All experiments were repeated three times independently using cells from three different calves (*n* = 3 biological replicates). Statistical analysis was performed based on these biological replicates. The data are expressed as the mean ± SEM.

To investigate the effect of NEFAs on hepatocyte pyroptosis, hepatocytes were treated with NEFAs (0, 0.3, 0.6, 1.2, 2.4 mM) for 12 h. To explore the synergistic effect of NEFAs and inflammatory responses in hepatocyte pyroptosis, cells were pretreated with 1 μg/mL LPS (L8880, Solarbio, Beijing, China) for 6 h, followed by treatment with 1.2 mM NEFAs for an additional 12 h. The selection of 1.2 mM NEFAs was informed by our initial dose-response experiment (Figure 1), which showed it significantly induced pyroptosis markers, and its relevance to the elevated circulating NEFA levels (approx. 0.93 mM) measured in subclinical ketotic cows. The 1 μg/mL LPS dosage was chosen based on established literature protocols for inducing inflammatory stress in various bovine cell models. To further investigate the role of pyroptosis in NEFAs and inflammatory response-mediated liver injury, cells were pretreated with 1 μg/mL LPS (with or without Ac-YVAD-cmk or MCC950, both at 10 μM) for 6 h, followed by treatment with 1.2 mM NEFAs (with or without Ac-YVAD-cmk or MCC950, both at 10 μM) for 12 h. MCC950 and Ac-YVAD-cmk were procured from MedChemExpress (HY-18738, HY-12815A, Monmouth Junction, NJ, USA).

### 2.3. Cell Viability Assay

Dairy cow hepatocytes and HepG2 cells were seeded in 96-well plates at a density of 5 × 10^3^ cells per well. Cell viability was determined using a Cell Counting Kit-8 (CCK-8) Kit (CA1210, Solarbio, Beijing, China). The absorbance of each well was measured at 450 nm using a microplate reader (BioTek, Winooski, VT, USA). The kit demonstrated high reproducibility, with intra-assay coefficient of variation (CV) less than 5% and inter-assay CV less than 10%.

### 2.4. Quantitative Real-Time PCR

Total RNA was extracted from hepatocytes using the SteadyPure RNA Extraction Kit (AG21102, Accurate Biology, Changsha, Hunan, China) according to the manufacturer’s instructions. RNA concentration and purity were measured using a Nanodrop (Thermo Fisher Scientific, Waltham, MA, USA). The A260/A280 ratio of the extracted RNA was maintained between 1.8 and 2.0, in accordance with the Minimum Information for Publication of Quantitative Real-Time PCR Experiments (MIQE) guidelines [21]. Subsequently, cDNA was generated using an Evo M-MLV reverse transcription kit (AG11728, Accurate Biology, Changsha, Hunan, China) following the manufacturer’s protocol. qRT-PCR was conducted using a SYBR Green Premix Pro TaqHS qPCR Kit (AG11701, Accurate Biology, Changsha, Hunan, China) on a Bio-Rad CFX96™ Real-Time PCR Detection System (Bio-Rad Laboratories, Hercules, CA, USA). The qRT-PCR conditions were as follows: initial denaturation at 95 °C for 30 s, followed by 40 cycles of 95 °C for 5 s and 60 °C for 30 s. The sequences of the primers used for PCR are listed in Table S1. The relative gene expression was normalized to both ACTB and GAPDH, and data analysis was performed using the 2^−ΔΔCt^ method.

### 2.5. Western Blotting

Hepatocytes were washed twice with ice-cold PBS. Total protein was extracted from cells using RIPA lysis buffer (C1055, Applygen, Beijing, China), and protein concentrations were measured using a BCA protein assay kit (P1511, Applygen, Beijing, China). Equal aliquots of protein were loaded and separated by 12% SDS-PAGE gels, then transferred onto polyvinylidene difluoride (PVDF) membranes. The membranes were blocked with 3% or 5% BSA for 2 h at room temperature, then probed with primary antibodies against NLRP3 (Cat. No. 19771-1-AP, 1:500), ASC (Cat. No. 10500-1-AP, 1:1000), Caspase-1/p20/p10 (Cat. No. 22915-1-AP, 1:3000), GSDMD (at. No. 20770-1-AP, 1:2000), and β-actin (Cat. No. Ab8226, 1:2000) overnight at 4 °C. After three washes with TBST, HRP-conjugated secondary antibodies (Cat. No. SA00001-1/Cat. No. SA00001-2, 1:2000) were added and incubated with the membranes for 45 min at room temperature. All antibodies were bought from Proteintech (Wuhan, Hubei, China). β-actin was used as a protein loading control. Immunoblot signals were detected using an ECL luminescence reagent (4AW011-100, 4A Biotech, Beijing, China) with a ChemiDoc XRS imaging system (Bio-Rad Laboratories, Hercules, CA, USA).

### 2.6. Collection and Fixation of Liver Samples

To further test whether assembly of the NLRP3 inflammasome took place in vivo, we used liver samples from healthy cows and cows with ketosis for immunofluorescence assays. All animals were housed under similar management conditions and sample collection was performed in accordance with protocols approved by the Institutional Animal Care and Use Committee (2021084). Liver tissue samples were obtained from dairy cows diagnosed with subclinical ketosis (blood β-hydroxybutyrate concentration within 1.2–3.0 mM but free of comorbidities by veterinary inspection) and from healthy control cows (β-hydroxybutyrate < 1.0 mmol/L, no symptoms), matched for age, parity, and lactation stage. Six healthy cows and six cows with subclinical ketosis were chosen from the same dairy farm located in Dingzhou, Hebei Province, China; their blood NEFA concentrations averaged 0.47 mM and 0.93 mM, respectively. Liver biopsy was performed using a stainless-steel trocar (K0088, Kelibo Animal Husbandry Technology Co., Ltd., Wuhan, China) with specifications of 31 cm in length and 6 mm in outer diameter. One random sample was selected in each group to perform immunofluorescence staining as a representative qualitative and quantitative assessment of NLRP3 inflammasome assembly in vivo. The selected samples were immediately fixed in 4% (*w*/*v*) paraformaldehyde solution (prepared in phosphate-buffered saline, pH 7.4) and stored at 4 °C for 24 h to ensure complete tissue fixation.

### 2.7. Immunofluorescence Assay

Both paraffin-embedded liver sections and cultured hepatocytes grown on glass coverslips were permeabilized with 0.2% Triton X-100 on ice for 10 min, and subsequently blocked with 5% BSA for 1 h. The cells were then incubated with primary antibodies, anti-NLRP3 (Proteintech, Wuhan, China; Cat. No. 19771-1-AP; 1:50) and anti-Caspase-1 (Proteintech, Cat. No. 22915-1-AP, 1:10), overnight at 4 °C. This was followed by incubation with appropriate secondary antibodies (Proteintech, Cat. No. SA00001-1/SA00001-2, 1:400) for 50 min at room temperature. Cell nuclei were counterstained with 4′,6-diamidino-2-phenylindole (DAPI). Fluorescence images were obtained using a fluorescence microscope (ZEISS Axio Vert.A1, Carl Zeiss Microscopy GmbH, Oberkochen, Germany).

### 2.8. Measurements of Caspase-1 Activity and IL-18 and IL-1β Concentrations

Caspase-1 activity was assayed using the FAM-FLICA Caspase-1 Assay Kit (#97, ImmunoChemistry Technologies, Bloomington, MN, USA) according to the manufacturer’s instructions. Fluorescence was measured using a fluorescence microscope with excitation/emission wavelengths of 488/520 nm. The results of Caspase-1 activity and Propidium Iodide (PI) staining were expressed as relative fluorescence intensity. For each independent experiment, the average fluorescence intensity of the treatment groups was divided by the average intensity of the control group to determine the fold change. Specifically, for PI staining, the number of positive cells was quantified from three randomly selected microscopic fields per well. The data were presented as the average count per field and normalized relative to the total cell count. The concentrations of IL-18 and IL-1β were measured using their respective enzyme-linked immunosorbent assay (ELISA) kits (AF4043-A, AF4049-A, AiFang biological, Changsha, Hunan, China) according to the manufacturer’s instructions. The intra-assay CV for the IL-1β and IL-18 ELISA kits was less than 10% and the inter-assay CV was less than 15%.

### 2.9. Statistical Analysis

Statistical analyses were conducted using SPSS software version 27.0 (IBM, Chicago, IL, USA) and GraphPad Prism version 8.0 (GraphPad Software, San Diego, CA, USA). All statistical comparisons were performed using the means of three independent biological replicates (*n* = 3). For each biological replicate, three technical replicates were conducted to ensure internal consistency. Data are expressed as means ± standard error of the means (SEM). Data were analyzed using one-way ANOVA with a subsequent Bonferroni correction. A *p*-value of <0.05 was considered to indicate a statistically significant difference.

## 3. Results

### 3.1. NEFA-Induced Pyroptosis in Bovine Hepatocytes

In calf hepatocytes, the mRNA expression of *NLRP3*, *CASP1*, *GSDMD*, *IL18*, and *IL1B* was upregulated in a dose-dependent manner with the increase in NEFA concentrations, particularly at 1.2 mM (Figure 1A–E, *p* ≤ 0.038). Western blot results were consistent with the qPCR results. Protein expression levels of NLRP3, ASC, Caspase-1, and GSDMD also demonstrated a linear increase with NEFA dosages (Figure 1F–J, *p* ≤ 0.045).

### 3.2. NEFA-LPS Synergy-Induced Pyroptosis in Bovine Hepatocytes

**Figure 2:**
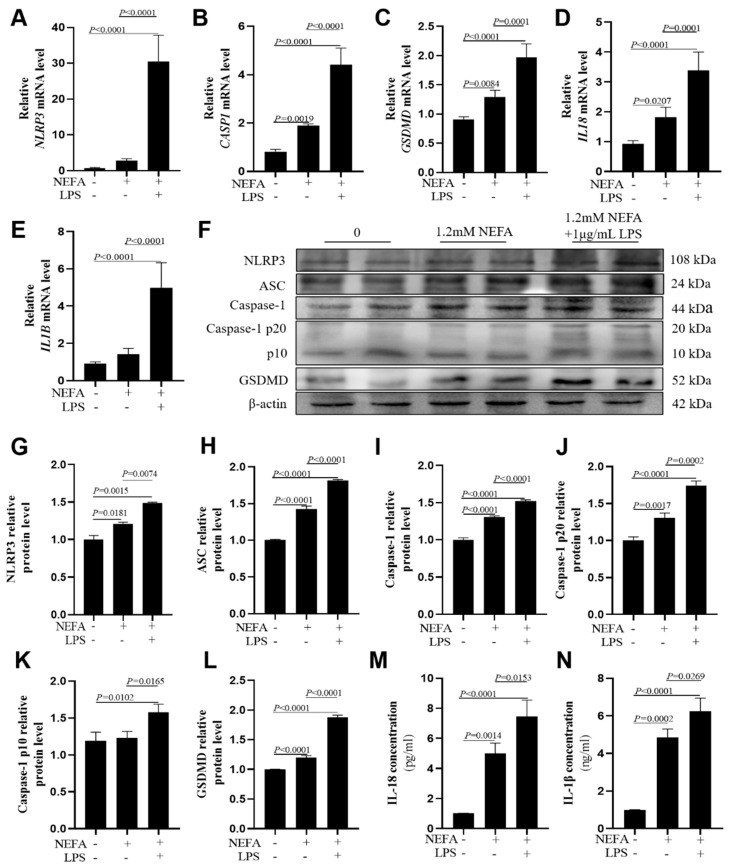
Effects of NEFAs plus LPS treatments on the expression of NLRP3, Caspase-1, GSDMD, IL18 and IL1B in dairy cow hepatocytes. (**A**–**E**) The mRNA relative expression of *NLRP3*, *CASP1*, *GSDMD*, *IL18* and *IL1B* was measured via real-time quantitative PCR with *ACTB* and *GAPDH* as the house keeping genes. (**F**–**L**) The relative protein levels of NLRP3, ASC, Caspase-1, Caspase-1 p20, p10 and GSDMD was measured via western blotting. (**M**,**N**) Concentrations of IL-1β and IL-18 in culture supernatants were determined using ELISA kits. All experiments were repeated three times independently using cells from three different calves (*n* = 3 biological replicates). Statistical analysis was performed based on these biological replicates. The data are expressed as the mean ± SEM.

In bovine hepatocytes, compared with the NEFA group, the mRNA abundance of *NLRP3*, *CASP1*, *GSDMD*, *IL18*, and *IL1B* was greater in the NEFA + LPS group (Figure 2A–E, *p* ≤ 0.0001). Western blot analysis of NLRP3, ASC, Caspase-1, cleaved Caspase-1(p20/p10), and GSDMD protein levels confirmed these observations (Figure 2F–L, *p* ≤ 0.0165). IL-18 and IL-1β concentrations in cell culture media were greater in the NEFA + LPS group than the NEFA group (Figure 2M,N, *p* ≤ 0.0269). Similar results were also obtained in HepG2 cells repeating the aforementioned experiments (Figure S1).

### 3.3. NEFA-LPS Synergy Enhanced the Assembly of NLRP3 Inflammasome

**Figure 3:**
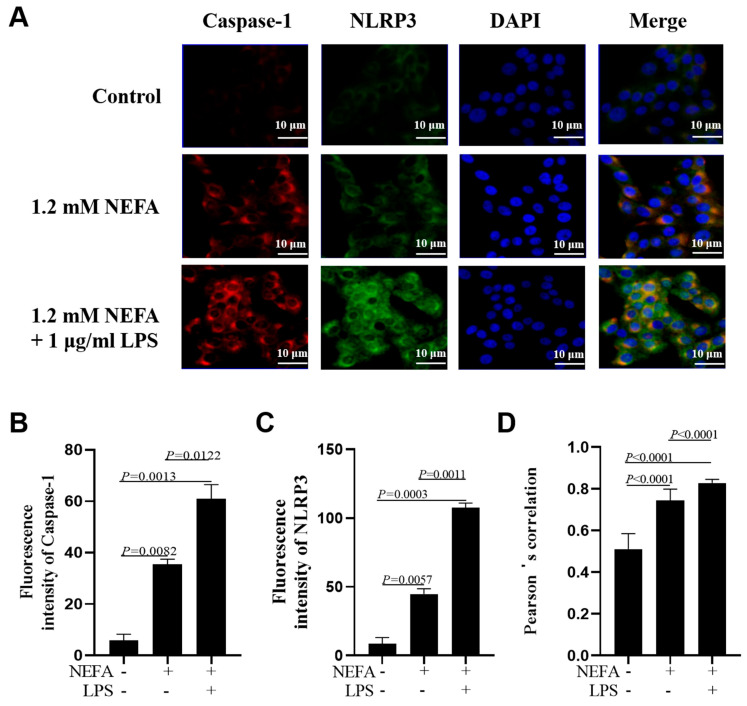
Effects of NEFAs plus LPS treatments on NLRP3 inflammasome assembly in dairy cow hepatocytes. (**A**) Immunofluorescence staining of DNA (blue), Caspase-1 (red) and NLRP3 (green) in dairy cow hepatocytes. (**B**,**C**) Fluorescence intensity of Caspase-1 and NLRP3. (**D**) Colocalization analysis of Caspase-1 and NLRP3. All experiments were repeated three times independently using cells from three different calves (*n* = 3 biological replicates). Statistical analysis was performed based on these biological replicates. The data are expressed as the mean ± SEM.

Immunofluorescence staining assays showed that the fluorescence intensity of NLRP3 and Caspase-1 was greater in bovine hepatocytes treated with NEFA + LPS than in the NEFA treatment alone (Figure 3A–C, *p* ≤ 0.0122). Pearson’s correlation analysis revealed that the co-localization of NLRP3 and Caspase-1 was also enhanced in the NEFA + LPS treatment (Figure 3D, *p* < 0.0001).

### 3.4. NEFA-LPS Synergy Led to Caspase-1 Activation

**Figure 4:**
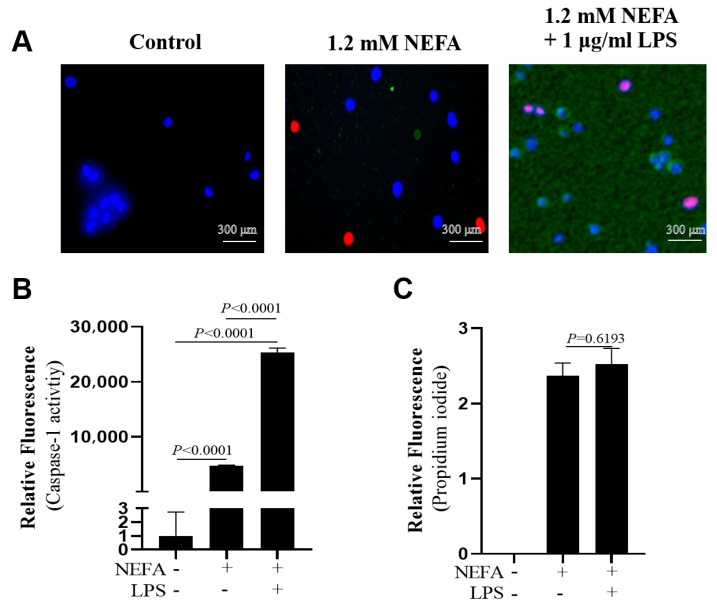
Effects of NEFAs plus LPS treatments on the activity of Caspase-1 in dairy cow hepatocytes. (**A**) Fluorescent staining of Caspase-1 (green). (**B**) Fluorescence intensity of Caspase-1. (**C**) Propidium iodide staining. All experiments were repeated three times independently using cells from three different calves (*n* = 3 biological replicates). Statistical analysis was performed based on these biological replicates. The data are expressed as the mean ± SEM.

Co-treatment with NEFAs and LPS led to greater Caspase-1 activity compared with NEFA treatment alone (Figure 4A,B, *p* < 0.0001). However, there was no difference regarding the number of PI-positive cells between the two groups (Figure 4A,C, *p* = 0.6193).

### 3.5. Both NLRP3 Inhibitor and Caspase-1 Inhibitor Suppressed NEFA + LPS-Induced Upregulation of Pyroptosis-Related Protein Levels

**Figure 5:**
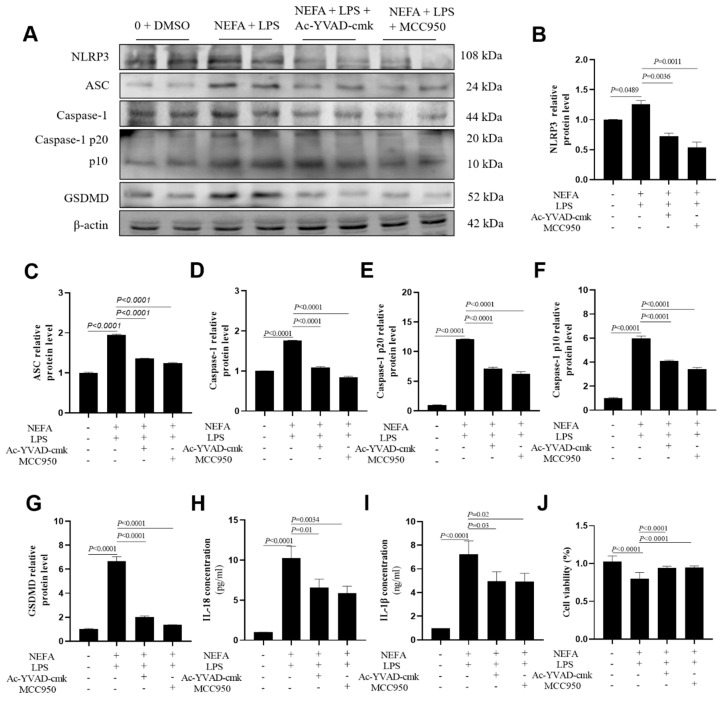
Effects of Caspase-1 inhibitor and NLRP3 inhibitor on the expression of NLRP3, ASC, Caspase-1, Caspase-1 p20, Caspase-1 p10, GSDMD, IL-18 and IL-1B in dairy cow hepatocytes. (**A**–**G**) The relative protein expression of NLRP3, ASC, Caspase-1, Caspase-1 p20, Caspase-1 p10 and GSDMD against the reference protein β-actin. (**H**,**I**) Levels of IL-1β and IL-18 in culture supernatants was determined using ELISA kits. (**J**) Cell viability of dairy cow hepatocytes treated with Ac-YVAD-cmk or MCC95. All experiments were repeated three times independently using cells from three different calves (*n* = 3 biological replicates). Statistical analysis was performed based on these biological replicates. The data are expressed as the mean ± SEM.

Preliminary experiments indicated that treatment with MCC950 or Ac-YVAD-cmk alone (10 μM) had no significant effect on cell viability or the basal expression of pyroptosis-related markers in the absence of NEFAs and LPS. Consequently, both MCC950 and Ac-YVAD-cmk incubations downregulated pyroptosis-related protein levels, namely, NLRP3, ASC, Caspase-1, cleaved Caspase-1(p20/p10), and GSDMD (Figure 5A–G, *p* ≤ 0.0036). The concentrations of IL-18 and IL-1β in cell culture media were lower after MCC950 and Ac-YVAD-cmk incubation (Figure 5H,I, *p* ≤ 0.03).

Similar results were also obtained in HepG2 cells after repeating the experiments (Figure S2). Both bovine hepatocytes and HepG2 cells displayed greater cell viability after addition of MCC950 and Ac-YVAD-cmk (Figure 5 and Figure S2).

### 3.6. Both NLRP3 Inhibitor and Caspase-1 Inhibitor Suppressed NEFA + LPS-Induced Assembly of NLRP3 Inflammasome

**Figure 6:**
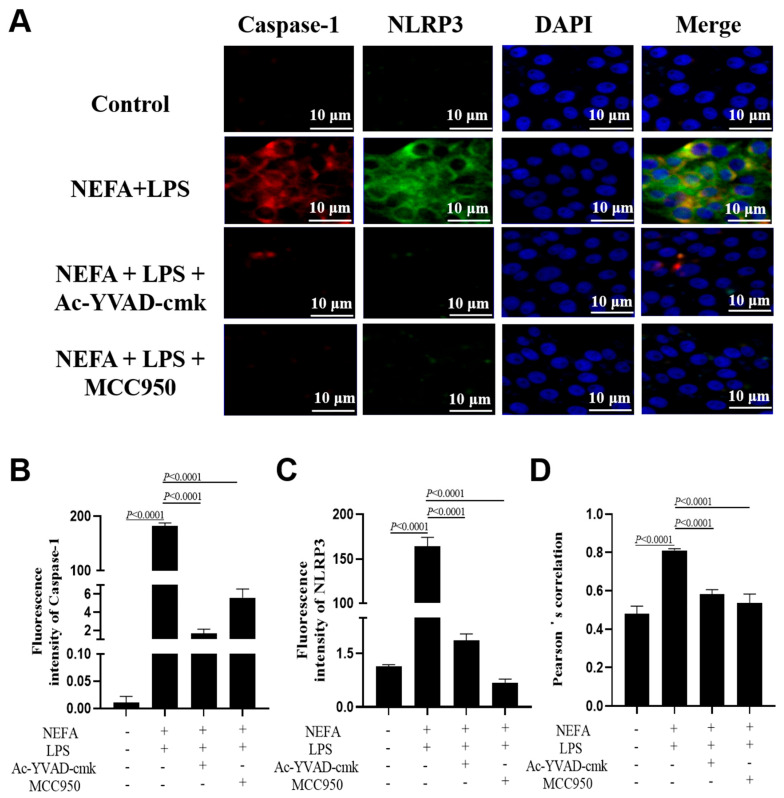
Effects of Caspase-1 inhibitor and NLRP3 inhibitor on the assembly of NLRP3 inflammasome in dairy cow hepatocytes. (**A**) Immunofluorescence staining of DNA (blue), Caspase-1 (red) and NLRP3 (green). (**B**,**C**) Fluorescence intensity of Caspase-1 and NLRP3. (**D**) Colocalization analysis of Caspase-1 and NLRP3. All experiments were repeated three times independently using cells from three different calves (*n* = 3 biological replicates). Statistical analysis was performed based on these biological replicates. The data are expressed as the mean ± SEM.

A similar reversal effect was observed in the immunofluorescence results obtained from bovine hepatocytes with incubation with the NLRP3 and the Caspase-1 inhibitor. Specifically, both the fluorescence intensity of NLRP3 and Caspase-1 (Figure 6A–C, *p* < 0.0001) and their co-localization (Figure 6D, *p* < 0.0001) were lower in the cells treated with MCC950 or Ac-YVAD-cmk.

### 3.7. Both NLRP3 Inhibitor and Caspase-1 Inhibitor Suppressed NEFA + LPS-Induced Caspase-1 Activation

**Figure 7:**
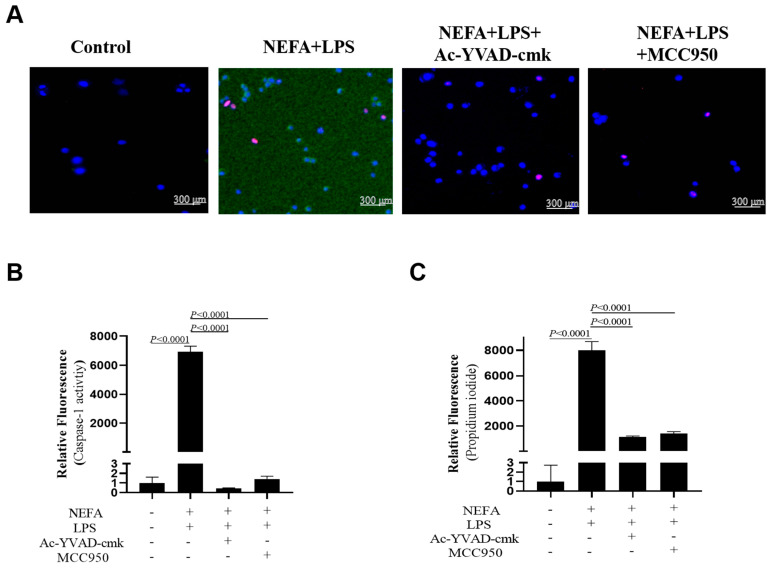
Effects of Caspase-1 inhibitor and NLRP3 inhibitor on activity of Caspase-1 in dairy cow hepatocytes. (**A**) Fluorescent staining of Caspase-1 (green). (**B**) Fluorescence intensity of Caspase-1. (**C**) Propidium iodide staining. All experiments were repeated three times independently using cells from three different calves (*n* = 3 biological replicates). Statistical analysis was performed based on these biological replicates. The data are expressed as the mean ± SEM.

As shown in Figure 7A,B, Caspase-1 activity was reduced in bovine hepatocytes after MCC950 and Ac-YVAD-cmk incubation (*p* < 0.0001). Furthermore, as indicated by PI staining, the number of dead cells also was lower (Figure 7A,C, *p* < 0.0001).

### 3.8. Hepatic Assembly of NLRP3 Inflammasome Took Place in Vivo

**Figure 8:**
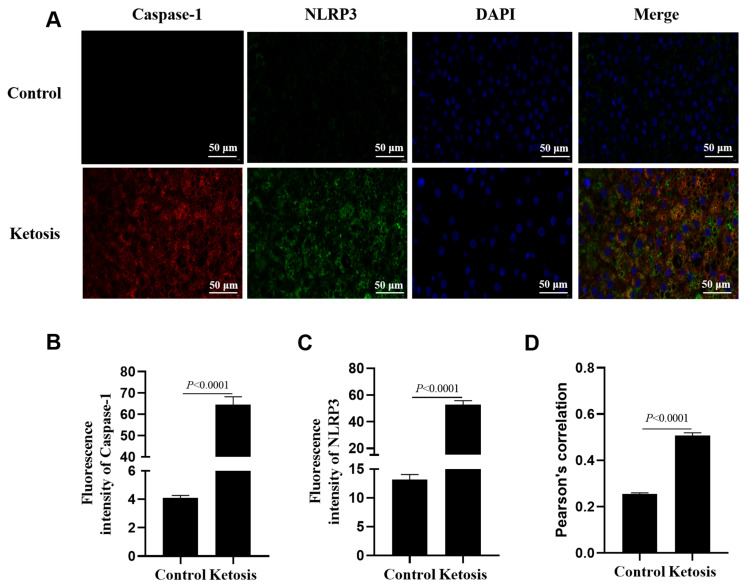
Immunofluorescence assays for liver sections from a representative subclinical ketotic cow and a representative healthy cow. (**A**) Immunofluorescence staining of DNA (blue), Caspase-1 (red) and NLRP3 (green). (**B**,**C**) Fluorescence intensity of Caspase-1 and NLRP3. (**D**) Colocalization analysis of Caspase-1 and NLRP3. Data in panels B-D represent the mean ± SEM of three random microscopic fields from these individual samples.

To provide visual evidence of in vivo inflammasome assembly, there was a marked increase in the fluorescence intensity of NLRP3 and Caspase-1 in a representative liver tissue section from a subclinical ketotic cow compared to a healthy control; the sick cow possessed an enhanced co-localization of NLRP3 and Caspase-1 in the liver (Figure 8, *p* < 0.0001). Statistical quantification for this figure was based on the analysis of three independent microscopic fields from each representative section.

## 4. Discussion

Elevated circulating NEFAs and LPS co-occur during the transition period [3]. The liver is an important organ for the disposal of both NEFAs and LPS. However, the synergistic effects of NEFAs and LPS have seldom been investigated in bovine hepatocytes. We hypothesized that NEFAs synergize with LPS to promote pyroptosis in hepatocytes. We have tested this hypothesis in bovine hepatocytes and HepG2 cells. The results of the present study supported this hypothesis, thereby offering a potential therapeutic target to prevent or alleviate liver injury in dairy cows during the transition period.

Pyroptosis is a type of programmed cell death closely related to inflammation, and it is a major contributor to hepatic injury in rodents and humans [22,23]. In bovine hepatocytes, our data indicated that pyroptosis is involved in NEFA-induced hepatocyte death, as supported by the following evidence. First, the expression levels of NLRP3, CASP1, GSDMD, IL18, and IL1B were upregulated in response to NEFAs in a dose-dependent manner. Cows with moderate fatty liver possess higher blood NEFA concentrations, and hepatic protein levels of NLRP3 and Caspase-1 are upregulated compared with healthy controls; calf hepatocytes treated with 1.2 mM NEFAs display greater NLRP3, Caspase-1 and IL1B levels [24], which are consistent with our observations. Furthermore, immunofluorescence assays revealed that the colocalization of NLRP3 and Caspase-1 was enhanced upon 1.2 mM NEFA challenge. The canonical NLRP3 inflammasome is composed of NLRP3, ASC, and pro-Caspase-1. When sensing certain signals, this complex assembles and promotes the auto-cleavage and activation of Caspase-1. Thus, our data suggested that high NEFAs resulted in the assembly of the NLRP3 inflammasome. Third, IL-1β and IL-18 concentrations in cell media were greater upon 1.2 mM NEFA incubation. IL-18 and IL-1β serve as critical signaling nodes within the NLRP3 inflammasome pathway. Activation of the NLRP3 inflammasome leads to the activation of Caspase-1 and the subsequent release of IL-18 and IL-1β, and this release will further initiate pyroptosis [25]. Our results that free fatty acids cause pyroptosis in hepatocytes are further supported by observations in rodents and human cells. The expression levels of NLRP3, N-GSDMD, cleaved-Caspase-1, and mature IL-1β in the liver were greater in HFD-group mice compared with controls [26]. Protein levels of NLRP3, Caspase-1 (p10), IL-1β, and GSDMD-N were markedly increased in palmitic acid-induced HepG2 cells [27].

Bacterial LPS aggravated the toxic effect of high NEFA levels on bovine hepatocytes. Indeed, LPS alone can induce NLRP3 inflammasome activation in mouse primary hepatocytes and in a non-alcoholic steatohepatitis mouse model [28]. Here, we used 1 μg/mL LPS to stimulate bovine hepatocytes, a similar dose that others adopted to treat multiple types of bovine cells [29,30,31,32]. At this dose, LPS exposure in addition to NEFAs challenge upregulated the expression of NLRP3, CASP1, GSDMD, IL18, and IL1B. Also, the assembly of NLRP3 was enhanced as indicated by the immunofluorescence staining. The release of mature IL-18 and IL-1β into cell media was promoted with the addition of LPS as well. The synergistic induction of pyroptosis by NEFAs and LPS likely involves a two-signal mechanism [33]. The observed upregulation of NLRP3, IL18, and IL1B mRNA abundance indicates that NEFAs and LPS provide the ‘priming’ signal, likely through the activation of the TLR4/NF-κB pathway, which is a common receptor for both endotoxins and saturated fatty acids [34,35]. Following priming, the ‘activation’ signal promotes the assembly of the NLRP3-ASC-Caspase-1 complex. In bovine hepatocytes, this second signal might be triggered by NEFA-induced mitochondrial dysfunction or the production of reactive oxygen species (ROS), both of which are potent activators of the NLRP3 inflammasome [36]. Our results using specific inhibitors (MCC950 and Ac-YVAD-cmk) further confirm that the downstream execution of this signaling remains dependent on the assembly and catalytic activity of the NLRP3 inflammasome. In alignment with our results, isolated hepatocytes from C57Bl/6 mice on a normal rodent diet were treated with palmitic acid (PA, 0.33 mM), LPS (1 μg/mL), or both for 6 h, and Caspase 1 activity and IL-1β levels were the highest in the PA + LPS group [37]. In vivo assay also confirmed that hepatic assembly of the NLRP3 inflammasome took place in the subclinical ketotic cow. However, it should be acknowledged that the in vivo evidence in this study was derived from representative samples analyzed through IF. Due to the limited volume of liver tissue obtained via trocar biopsy, which prioritized histological integrity for co-localization analysis, further quantification of pyroptosis markers through Western blotting or qPCR was not feasible. While these findings are preliminary and correlative in nature, the association between systemic metabolic stress and hepatic pyroptosis is further supported by the alignment of blood parameters with tissue markers. Specifically, the elevated circulating NEFA and BHB levels in ketotic cows coincided with the intensified hepatic activation of NLRP3 and Caspase-1. This observation is consistent with our in vitro findings, where NEFAs induced the expression of pyroptosis-related markers in a dose-dependent manner, particularly at concentrations mimicking the severe NEB observed in clinical cases. Consequently, these results confirm that LPS synergizes with NEFAs to promote the assembly of the NLRP3 inflammasome and further activate Caspase-1 to cause pyroptosis in bovine hepatocytes.

The induction of pyroptosis in hepatocytes has profound functional consequences on the metabolic stability of transition cows. Beyond being a mode of cell death, pyroptosis releases mature IL-1β and IL-18 into the hepatic microenvironment [38]. These cytokines can directly impair hepatic gluconeogenesis and inhibit fatty acid oxidation, two processes that are vital for managing the NEB characteristic of ketosis [39,40]. Furthermore, the physical destruction of hepatocytes through GSDMD-mediated pore formation reduces the overall metabolic capacity of the liver to process circulating NEFAs and export triglycerides [41]. Consequently, hepatocyte pyroptosis may not only be a result of metabolic stress but also a driver of hepatic metabolic failure, potentially creating a vicious cycle that accelerates the progression from subclinical to clinical ketosis.

Our observations, though mostly in vitro, further provide insights into the health management of dairy cows during the transition period. Our findings elucidate a previously unrecognized synergy between metabolic stress (high NEFA levels) and inflammatory stimuli (LPS) in driving hepatocyte loss through the NLRP3-Caspase-1-GSDMD pathway. This mechanism suggests that maintaining hepatic health in transition cows requires a dual focus: minimizing negative energy balance and controlling systemic inflammation. By understanding these cellular pathways, future research can better target the prevention of liver injury, although the development of specific clinical interventions will require extensive in vivo validation beyond the scope of this study.

## 5. Conclusions

In summary, our study demonstrates that NEB-related concentrations of NEFAs and the endotoxin LPS synergistically trigger bovine hepatocyte pyroptosis. However, it must be noted that our in vivo findings in ketotic cows remain preliminary due to the limited sample size. Future research with larger cohorts and functional interventions is required to confirm these observations and establish a definitive causal link between hepatocyte pyroptosis and the clinical progression of ketosis.

## Data Availability

The original contributions presented in this study are included in the article/Supplementary Materials. Further inquiries can be directed to the corresponding author.

## Supplementary Materials

The following supporting information can be downloaded at: https://www.mdpi.com/article/10.3390/metabo16010053/s1, Figure S1: Effects of NEFA plus LPS treatments on the expression of NLRP3, Caspase-1, GSDMD, IL18 and IL1B in HepG2 cells. A-E The mRNA relative expression of *NLRP3*, *CASP1*, *GSDMD*, *IL18* and *IL1B* was measured via real-time quantitative PCR; *ACTB* and *GAPDH* were selected as the reference genes. F-L The relative protein expression of NLRP3, ASC, Caspase-1, Caspase-1 p20, Caspase-1 p10, GSDMD against the reference protein β-actin; Figure S2: A-G Effects of Caspase-1 inhibitor and NLRP3 inhibitor on protein abundance of NLRP3, ASC, Caspase-1, Caspase-1 p20, Caspase-1 p10 and GSDMD in HepG2 cells. H Cell viability of HepG2 cells treated with Ac-YVAD-cmk or MCC95; Table S1: List of primer sequences of target genes for real-time quantitative PCR.

## Abbreviations

The following abbreviations are used in this manuscript:

NEB	negative energy balance
NEFA	non-esterified fatty acid
ROS	reactive oxygen species
LPS	lipopolysaccharide
TNF-α	tumor necrosis factor-α
IL-1β	interleukin-1β
IL-6	interleukin-6
GSDMD	gasdermin D
FBS	fetal bovine serum
BSA	bovine serum albumin
CCK-8	Cell Counting Kit-8
MIQE	Minimum Information for Publication of Quantitative Real-Time PCR Experiments PVDF polyvinylidene difluoride
DAPI	4,6-diamidino-2-phenylindole
ELISA	enzyme-linked immunosorbent assay
SEM	standard error of the mean
PYD	pyrin domain
NSAID	nonsteroidal anti-inflammatory drugs
