# Supplementary material for: Free Fatty Acids and Endotoxins Synergically Induce Pyroptosis in Bovine Hepatocytes
Source: Metabolites. 2026 Jan 8;16(1):53. doi: 10.3390/metabo16010053 (PMC12844290; doi:10.3390/metabo16010053)
Supplement: Supplementary file 1 [file metabolites-16-00053-s001.zip › Table S1.pdf]

Table S1 List of primer sequences of target genes for real-time quantitative PCR

Gene	Species	Sequences (5’ to 3’)	Length (bp)	NCBI accession No.
ACTB	Bovine	F: TCACCAACTGGGACGACA	205	NM_173979
		R: GCATACAGGGACAGCACA		
GAPDH	Bovine	F: CACAGTCAAGGCAGAGAACG	108	NM_001034034
		R: TACTCAGCACCAGCATCACC		
IL1B	Bovine	F: AGGTGGTGTCGGTCATCGT	195	NM_174093
		R: GCTCTCTGTCCTGGAGTTTGC		
IL18	Bovine	F: GATATGCCTGATTCTGACTG	122	XM_061440303.1
		R: GACATTTTCTTACACTGCACA		
NLRP3	Bovine	F: TGGGAGACTTCGGGATCAGACTTC	136	XM_059888315.1
		R: GTGGTTGGTGCTCAGGACAGATG		
CASP1	Bovine	F: AATAAATGGCTTGCTGGATGAG	267	XM_005215739.5
		R: CCTCCTGGTCCTGAAGATGC		
GSDMD	Bovine	F: GGCTGGCGACCACTGACGACTA	415	NM_001046160.3
		R: TGCTCCTGCCACGGGCTGCTTT		
ACTB	Human	F: CATGTACGTTGCTATCCAGGC	250	NM_001101.5
		R: CTCCTTAATGTCACGCACGAT		
GAPDH	Human	F: ACAACTTTGGTATCGTGGAAGG	93	NM_001256799.3
		R: GCCATCACGCCACAGTTTC		
IL1B	Human	F: ATGGCTTATTACAGTGGCAATGAGG	136	NM_000576.3
		R: AGTGGTGGTCGGAGATTCGTAG		
IL18	Human	F: ATCGGCCTCTATTTGAAGATATG	123	NM_001243211.2
		R: TCACAGAGATAGTTACAGCCATACC		
NLRP3	Human	F: CCACAAGATCGTGAGAAAACCC	91	NM_001079821.3
		R: CGGTCCTATGTGCTCGTCA		
CASP1	Human	F: CAGACAAGGGTGCTGAACAA	98	NM_001257119.3
		R: CGGAATAACGGAGTCAATCA		
GSDMD	Human	F: GCTTCCACTTCTACGATGCCATG	83	NM_024736.7
		R: GCCTGCGATCTTTGCCTGTC		
