# Supplementary figures and images for: Free Fatty Acids and Endotoxins Synergically Induce Pyroptosis in Bovine Hepatocytes
Source: Metabolites. 2026 Jan 8;16(1):53. doi: 10.3390/metabo16010053 (PMC12844290; doi:10.3390/metabo16010053)

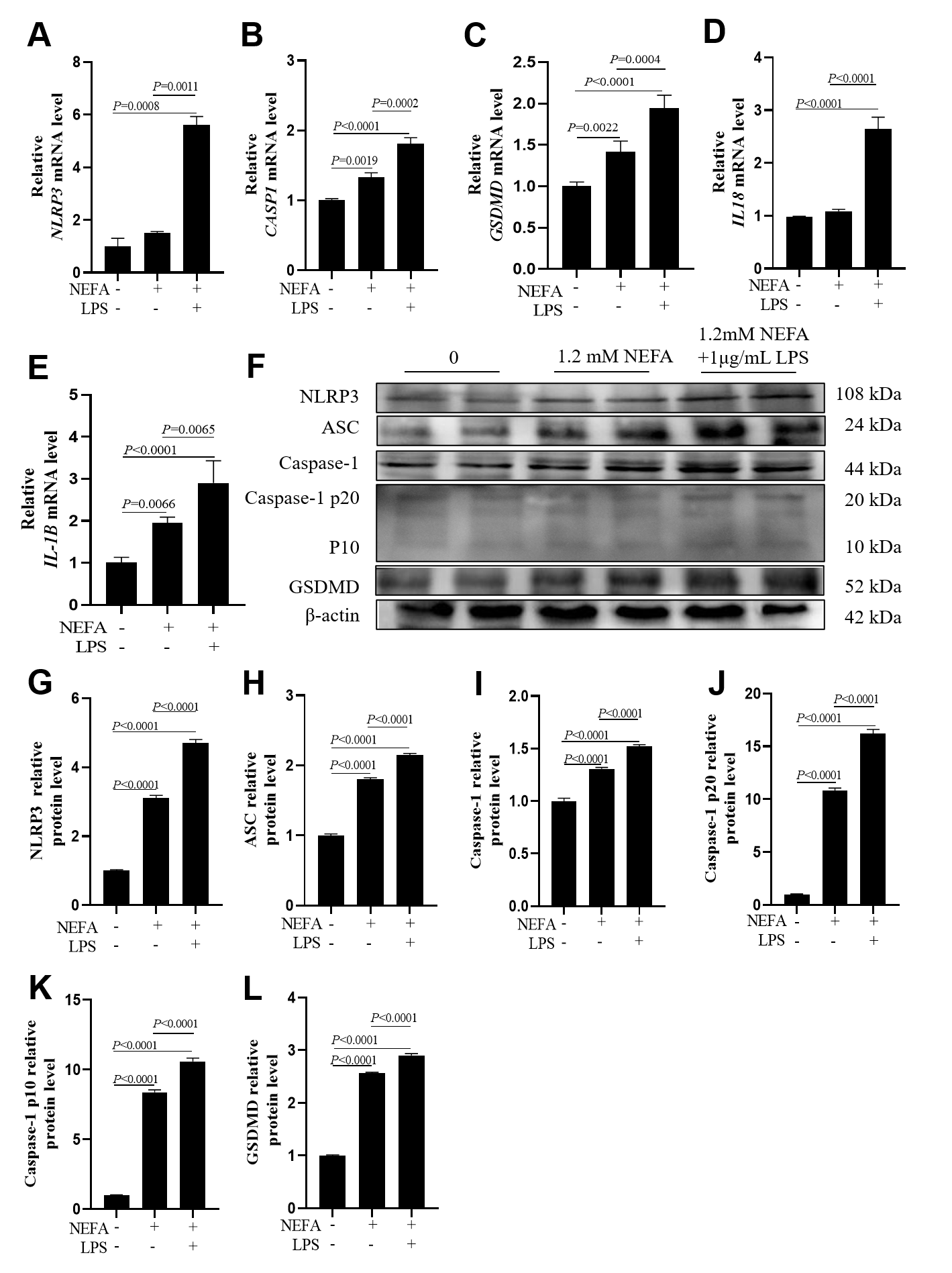

Supplement: Supplementary file 1 [file metabolites-16-00053-s001.zip › Figure S1.tif]

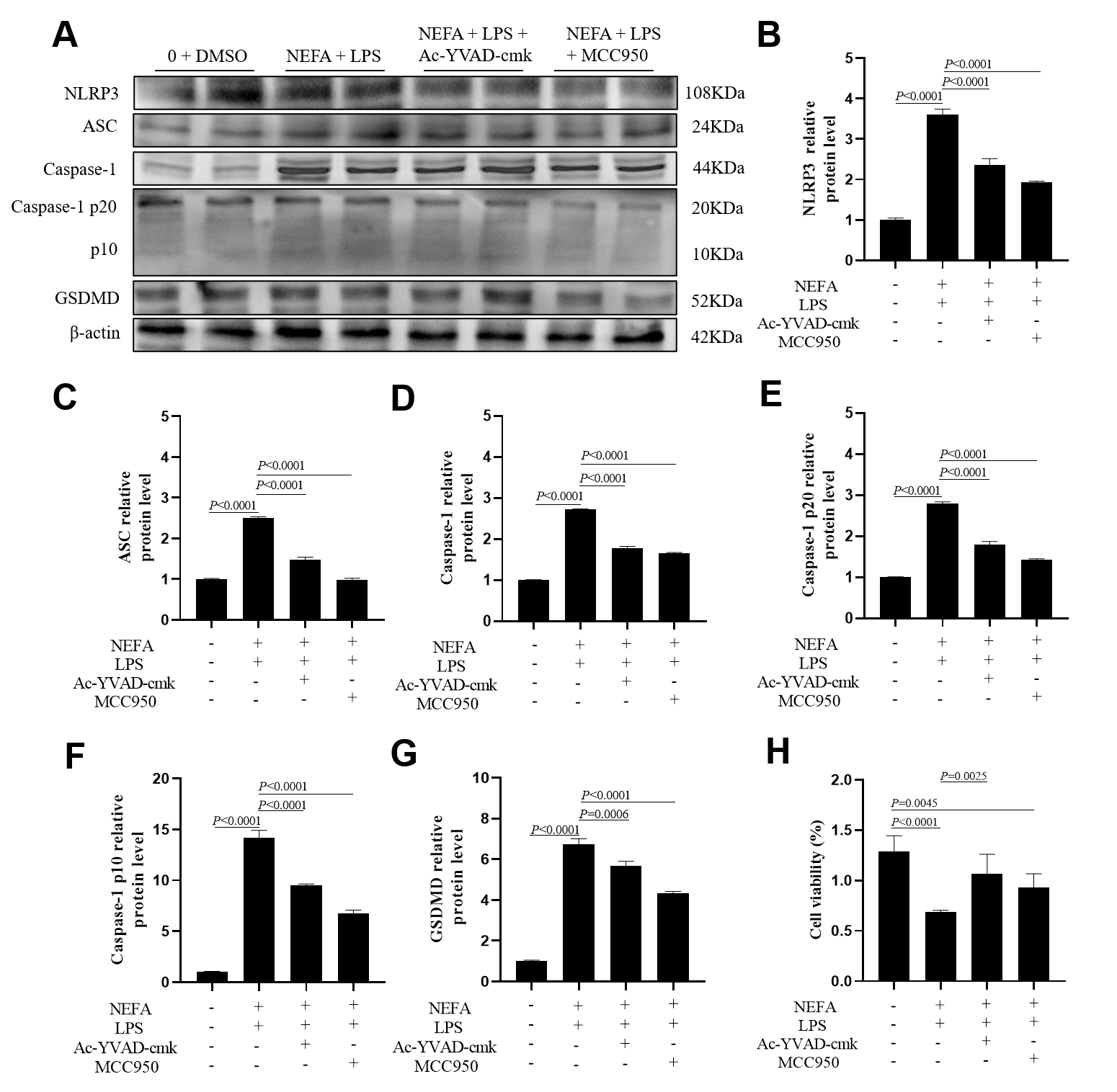

Supplement: Supplementary file 1 [file metabolites-16-00053-s001.zip › Figure S2.tif]
